# Evaluation of a virtual, simulated international public health peer-to-peer exchange learning experience

**DOI:** 10.3389/fpubh.2023.1144716

**Published:** 2023-04-13

**Authors:** Danish Ahmad, Rosemary A. McFarlane, Jennifer Smith, Deepak Saxena, Shawn Somerset, Dileep Mavalankar

**Affiliations:** ^1^School of Medicine and Psychology, College of Heath and Medicine, Australian National University, Canberra, ACT, Australia; ^2^Health Research Institute, University of Canberra, Canberra, ACT, Australia; ^3^Public Health Foundation of India, and Indian Institute of Public Health-Gandhinagar (IIPH-G), Gandhinagar, India; ^4^Faculty of Health, University of Canberra, Canberra, ACT, Australia; ^5^Learning and Teaching, University of Canberra, Canberra, ACT, Australia

**Keywords:** global health education, virtual learning and education environment, constructivist learning approach, global health evaluation, cross-cultural learning and teaching

## Abstract

**Introduction:**

Public Health’s (PH) global rise is accompanied by an increasing focus on training the new generation of PH graduates in interdisciplinary skills for multisectoral and cross-cultural engagement to develop an understanding of commonalities in health system issues and challenges in multi-cultural settings. Online teaching modalities provide an opportunity to enhance global health skill development through virtual engagement and peer exchange. However, current teaching pedagogy is limited in providing innovative modes of learning global health issues outside of traditional classroom settings with limited modalities of evidence-informed implementation models.

**Methods:**

This study designed, implemented, and evaluated a novel global health online synchronous module as proof of concept that incorporated elements of virtual Practice-based learning (PBL) using a case study approach offered to currently enrolled public health students at the University of Canberra (UC) and a partnering public health university from India, the Indian Institute of Public Health Gandhinagar (IIPH-G). Using constructive learning theory and the Social Determinants of Health framework, four online sessions were designed and implemented in August–September 2022. Formal process and outcome evaluation using a quantitative adapted survey of the validated International Student Experience survey (IES) at session end and findings provided.

**Results:**

Over 100 participating public health students from Australia and India provided narrative feedback and quantitative responses from the adapted IES instrument across four key dimensions, namely “motivation,” “personal development,” intellectual development, and “international perspectives” reporting an overall high mean impact of 4.29 (out of 5) across all four themes seen together. In essence, the sessions supported students to explore global health issues from a different cultural perspective while developing intercultural communication skills and enhancing their global exposure in real-time.

**Discussions:**

This innovation, implemented as a proof of concept, provided evidence, and demonstrated the implementation feasibility of a flexible virtual integrated practice-based module that can supplement classroom teaching. It provides participating students with the opportunity to develop intercultural understanding and communication competence as well as support global mindedness by engaging with international peers around focused global health case studies.

## 1. Introduction: Background and rationale for the educational activity innovation

Public Health (PH) has gained global prominence in the health sector as a multidisciplinary field able to meet emerging global health challenges (1, 2). Despite regional differences in health outcomes, common global health challenges such as the reemergent viral pandemics, rising non-communicable diseases and health inequities form a core part of health system functions and health workforce responsibilities (3). Moreover, common global health goals set by the United Nations now provide a unique opportunity for global health professionals to collaborate, learn from programmatic experience, and advance health promotion activities based on local cultural contexts (3).

At the same time, global health’s rise is accompanied by an increasing focus on training the new generation of global and public health graduates in interdisciplinary skills for multisectoral and cross-cultural engagement in varied healthcare settings (2–4). While interdisciplinary skills allow students to appreciate the multiple influences on health and working in diverse healthcare settings, transnational PH experiences allow students to develop a cultural appreciation and an understanding of cultural commonalities and differences in health system issues (3, 5–7). Current teaching pedagogy, however, is limited in providing innovative modes of enhancing competency-based outcomes for contemporary global health issues in a traditional classroom setting, especially for postgraduate public health courses (1, 3, 7, 8). Conventional classroom teaching provides students with knowledge of global health challenges but without the benefit of real-time exchange and learning from peers in different global settings (5–9).

This, in many instances, limits the opportunity for students and future global health practitioners to develop applied skills in developing culturally appropriate, health promotion strategies specific to local contexts, as well as developing communication skills when part of culturally and linguistically diverse professional work environments. Moreover, learning designers note the crucial role of integrated practice-based learning in cross-cultural cooperative learning groups (9, 10) *via* facilitated and scaffolded learning in developing knowledge and skills.

One of the ways to foster authentic cross-cultural learning is to provide opportunities for peer-to-peer engagement. The adoption of cross-cultural learning has usually been fostered through in-person classroom sessions where social connections and communication are better leveraged among attending participants. Moreover, greater cultural immersion has conventionally been linked to study abroad programs that have also been the primary mode for international health system exposure (11, 12). An evaluation by Tran et al. (13) in Australia, for example, the Australian Government’s flagship university student (undergraduate only) travel and study abroad (in semester) grant, the New Colombo Plan (NCP), showed an increasing number of students reporting greater cultural appreciation of regional host countries post-program participation, along with an increase in students choosing to work in jobs “based in the (Indo-pacific) region” (13). While such study abroad programs advance student global student mobility, most operate from Government funded programs based *in* high-income Global North countries like Australia. While in-country immersion is effective, we recognize asymmetry in global learning for students in countries in which such opportunities are not readily available, as well as for most post-graduate students and even those students from high-income countries who may have time limitations, financial constraints, and family carer responsibilities.

Moreover, this conventional model of study abroad programs has largely been replicated in the online iteration with virtual internship programs over the period of COVID-19 travel restrictions (14–17). Virtual placements increasingly offered through third-party for-profit providers during this time demonstrated the potential to provide powerful transnational learning. However, a scan of available literature for implementation modalities for public health experiential learning and study abroad pedagogies experiences highlighted a lack of opportunities to embed skills-based global health learning outcomes (18–22). Thus, we sought to develop an equitable, cross-cultural peer learning opportunity. Tailored to our courses and universities’ own teaching and learning curriculum and outcome (23).

This study showcases a series of virtual authentic cross-cultural online learning exchange sessions called the “International Peer Exchange Learning Innovation, the University of Canberra” or I-PELICAN, for global health, including Master of Public Health students between an Australian and Indian University. The University of Canberra (UC) is a public-funded Australian university with a focus on allied health courses and a strong focus on enhancing student equity in university enrollment, and participation (24). UC’s student equity focus includes removing structural inequalities that act as barriers to university entry, and subsequent course completion which is evident by the Times Higher Education ranking which placed UC at #1 globally in reducing inequalities in 2022 (24).

As such, our aim was to provide UC’s diverse cohort of global health students in-semester with an international virtual authentic cross-cultural learning experience with an overseas participating university.

Thus, the proposed innovation was to develop I-PELICAN sessions as proof of concept as an adaptable online module, delivered over four weeks during the academic semester that integrated into an existing Global Health unit at UC and in-semester for the postgraduate public health course at the participating Indian University – the Indian Institute of Public Health-Gandhinagar (IIPH-G). IIPH-G is India’s first and largest autonomous public health university offering postgraduate public health degrees, advanced research, and capacity building. IIPH-G and UC’s pre-existing academic relationship was leveraged to advance this academic innovation which provided students from both countries the opportunity to develop a deeper appreciation of health system gaps, not only highlighting differences in cultural contexts but, importantly, also showcasing common health issues faced by a high-income and lower-middle-income country relating to health care access and equity. This bidirectional student exchange in real time included several innovative educational activities which would build cross-cultural, peer exchange competencies for participating students.

## 2. Pedagogical framework(s), pedagogical principles, competencies/standards underlying the educational activity

I-PELICAN’s online module design was informed by the pedagogical expertise of an educational designer at UC, who worked with the PH teaching team to design an online social constructivist learning environment (9, 10) using practice-based learning. This approach views learning as a process of interpreting, building, modifying, and understanding reality through social interactions with others *via* cooperative learning (9). A review by Thomas et al. (2014) suggests that social constructivist learning theories in knowledge translation are essential for best practice in health professionals (10).

Practise-based learning (PBL) using a case study approach allows students to translate classroom theory to real-world issues, promoting competency development (9–15). While different models of PBL demonstrate success, mentor-based PBL sessions have increasingly been adopted by PH universities with higher student success outcomes. Research also shows that experiential learning is valued by both students and employers alike from a graduate readiness perspective (7–10). Yet only a minority of universities offer PBL integrated into PH courses. A systematic review in 2021 reported only 40 graduate PH programs globally offering practice-based learning (1). Incorporating traditional PBL is further challenged by the rise of online delivery of PH units (1). While online delivery opens educational access to a wider cohort of students, the mode is limited in offering advantages linked to place based PBL sessions (15–17, 25, 26). Thus, the pedagogy guided the development of a new global health online synchronous module that incorporates elements of a virtual PBL which can be integrated within an existing semester unit.

Each of the four linked online tutorial sessions embedded constructivist learning theory using practice-based learning focussing on real-world cases such as COVID-19, to which a contemporary global health framework was applied, the Social Determinants of Health (SDH), to scaffold the session activities (27). The selection of the SDH framework allowed the incorporation of contemporary global health theory, supporting students to develop a greater appreciation of how cultural factors could influence health care seeking at multiple levels of societal influence. The mentors facilitated online sessions by prompting structured problem-solving and skill development using the SDH framework. This approach involved incorporating complementary theories and pedagogical design to structure sessions where authentic student-led peer exchange could occur in real-time and in a collaborative yet culturally sensitive environment. As these sessions were embedded in an ongoing global health unit at UC and in the MPH at IIPH-G, the learning objectives developed an understanding and application of cultural awareness using a contemporary global health framework for health promotion design that could be applied in different cultural and social contexts. The learning objectives were assessed during the final I-PELICAN session through students’ application of new learning in producing group-based E-posters focussing on contemporary global health issues as per the case study assigned to them. The E-posters provided visual evidence of this application of learning and were used as a presentation aid for the groups to share and reflect on their learning with the whole cohort.

Based on the notion that constructivism is an active process of knowledge constructing rather than acquiring knowledge, and facilitated instruction is a process of supporting that construction rather than communicating knowledge (28), the intention was to design a supportive online environment that fostered engagement through active learning strategies which encouraged peer to peer exchange for cross-cultural learning, supported by the mentorship of a team of cross-institutional academic facilitators.

To illustrate the pedagogical approach in action, we outline below the structure and intended outcomes for the virtual sessions, all of which were based on Zoom and used collaborative software including Padlet^1^ and Google Docs^2^ to enable equal access by both groups for real-time sharing and collaboration.

The first pilot session set learning expectations and introduced cultural practices that mark gatherings, such as an Acknowledgement of Country (Australia) and Lighting of the Lamp (India), with time for the facilitator and student introductions. Academics from both institutes modeled cross-cultural collaboration by working through the required activity in Zoom’s main room before students were sent in mixed UC-IIPH-G groups to breakout rooms to do the same activities under the guidance of a facilitator/mentor. Students were asked to bring a photo/image that could be used to share cultural insights, and this created rich discussions and enabled students to not only introduce each other’s lived experience but to learn about virtual collaborative software and create new knowledge, reporting back to the main room with the groups’ reflections. This also scaffolded students toward the next tutorial activity, which was to bring photos depicting cultural influences on health care-seeking behaviors.

For consistency and clarity purposes, the same structure was applied to all tutorial sessions, which included a plenary session where academics from both institutions “modeled” the activity for that session by applying the SDH framework to analyze the various case studies. This provided clarity on the learning expectations and objectives for the group work which followed in break-out rooms. Each tutorial ended with a presentation in the main room by each group and resulting in active reflection and collection of feedback from students and facilitators. The immediacy of this feedback provided the authors an opportunity to refine subsequent sessions which highlighted to students that their feedback was valued and acted upon.

The embedded real-time process evaluation included facilitator and student qualitative free text captured in a specific feedback template pre-designed using Padlet and provided to each group at each session’s end. These reflections and feedback informed the subsequent session delivery and design. The I-PELICAN session’s formal evaluation comprised a Quantitative Qualtrics survey based on an adapted International Education Survey (IES) administered at the end of session four and created E-posters as visual evidence of the application of learning. Separately, a written reflective component as part of an in-semester assessment was included for UC students only two weeks after the final session.

## 3. Pedagogical format and learning environment; learning objectives; and results.

Study authors from the Australian and Indian universities jointly codesigned, implemented and evaluated four online sessions for a mixed cohort of over 100 students from both universities in August and September 2022. These sessions were styled around conventional Australian university tutorials, which provide greater space for student engagement and interaction. The I-PELICAN tutorial sessions thereafter “sessions” were structured to facilitate peer-to-peer learning using a case study approach, with 10 online break-out groups, each supported by a facilitator/mentor (drawn from both institutions). The mentor’s role, who was a UC or IIPHG academic, was to facilitate a supportive online environment, encourage student interaction in the break-out rooms and help provide input as a collaborator if required by individual student groups.

### 3.1. Pedagogical format and the learning environment

Guided by the UC educational designer, academics from both institutions workshopped the suggested pedagogical framework and approaches, including practice-based learning (PBL) using a case study approach and active learning *via* cross-cultural cooperative learning groups (9). Several key considerations and anticipated challenges of taking this pedagogical approach were identified in the initial month-long design phase of module development, and strategies were developed to address these challenges, which were further refined over the delivery of the module. This led to an emphasis on session planning, facilitator pre-briefing sessions, teaching resource preparation, and immediate collection of student and facilitator feedback after each session with the view to refining subsequent sessions.

Anticipated challenges identified in these academic/facilitator pre-briefing workshops included possible issues with cross-cultural cooperative learning group dynamics that may result in reduced student participation and case study contributions. Strategies identified to address these challenges included communicating to students ahead of the session to be clear about the purpose and expectations, scheduling a pilot/training session for students and staff, role-modeling by academics and the use of effective questioning techniques and allocating a facilitator per group as a mentor and learning support.

### 3.2. Session plan and learning objectives.

The overall learning objectives were co-designed by UC and IIPH-G academics (see Illustration 1 below) and scaffolded from session to session to help students progressively build their understanding, confidence, and cross-cultural perspectives through the application of the SDH framework to related PH case studies.

ILLUSTRATION 1 I-PELICAN session plan and learning objectives.

**Table:** 

	Training (pilot) Session	Session #1	Session #2	Session #3
Tutorial Theme		**Understanding India and Australia’s COVID journey**		
Tutorial Focus	Orientation/Context Setting	**New Knowledge Development**	**Skill Development**	**Applied Focus**
Learning objectives	Welcome, introductions, and refinement	Develop an understanding of how cultural and societal factors influence the perception of healthcare-seeking attitudes in an Indian and Australian context	Learn about analytical frameworks for systematically approaching contemporary public health issues in an international context	Apply theory and real-life experience to develop a health system intervention to address a contemporary global health challenge
Pre-session activity	Elements from all three main tutorials were trialed	Bring two photographs peer exchange for a cultural introduction themed around “person, place and health care setting” in the local community	Collect interview insights from family/friends on COVID-19 behaviors, attitudes, and knowledge using prompts	Read two open-access case studies on Tuberculosis management in India and refugee health in Australia

**Figure 1:**
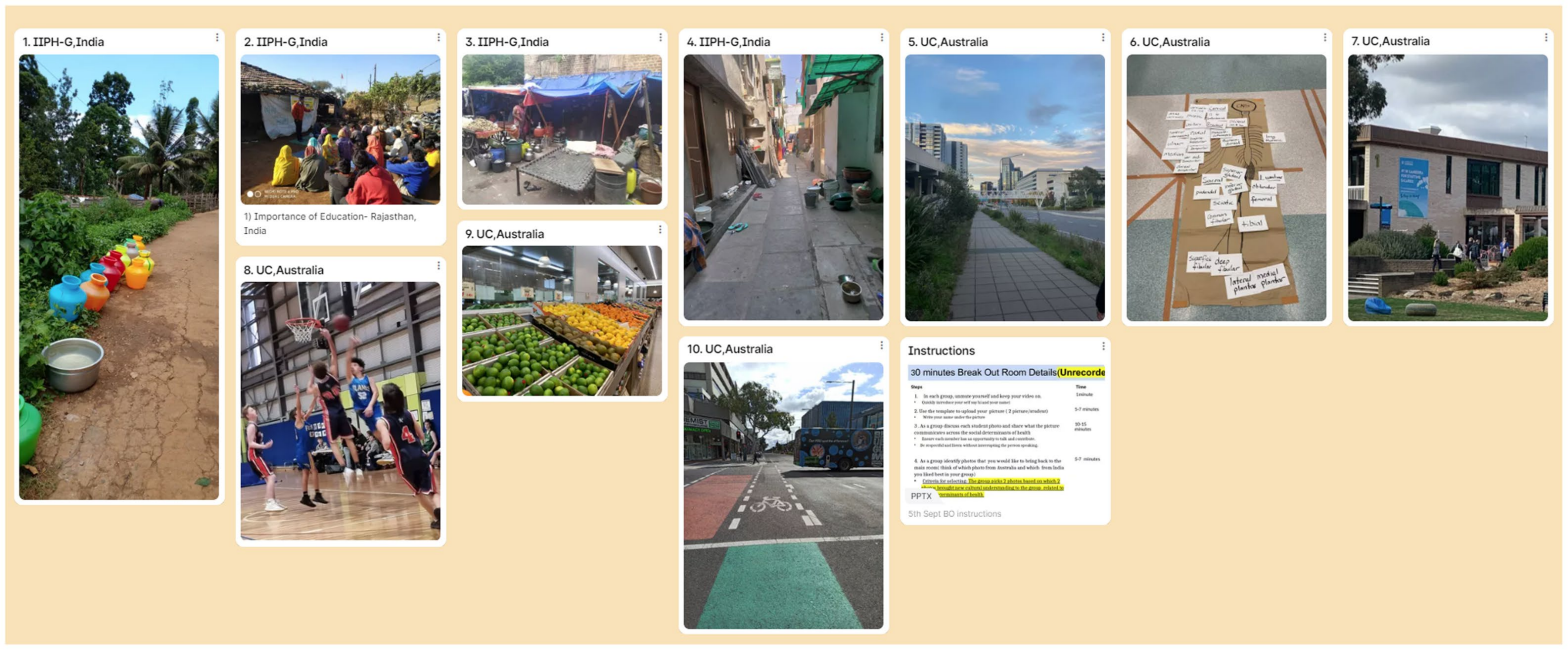
Shows cultural awareness group activity using photographs uploaded on Padlet in a Zoom breakout room in the first(pilot) I-PELICAN session.

To meet the learning objective of the **training (pilot)** session – welcome, introductions, and refinement students were briefed at each institution prior to the session, requesting students to source two photos/images that depicted culture in their context that students would be willing to share online to the broader cross-institutional group. Figure 1 below shows a filled template that has been reproduced with permissions for this study. This activity met the tutorial focus of context setting – orienting students to the overall module aims and making initial introductions in groups. Students were invited to share their initial feedback *via* Padlet at the end of the session.

With the focus of the first tutorial session being new knowledge development and to meet the learning objective of the session – to develop an understanding of how cultural and societal factors influence the perception of healthcare-seeking attitudes in an Indian and Australian context – students were again briefed to source and share two pictures each illustrating a disease problem in their country – using COVID-19 as the case study which enabled students to work from a shared experience. Again, this facilitated rich discussions, this time within a shared PH theoretical framework (SDH). Having identified cultural influences on healthcare-seeking behavior, students were required to prepare for tutorial #2 by collecting short interview insights from their family or friends regarding attitudes, behaviors, and knowledge about COVID-19 safe behaviors, vaccination, and post-COVID-19 perceptions.

In preparation for the **second tutorial session**, students were required to collect short interview insights from their family or friends regarding attitudes, behaviors and knowledge about COVID-19 safe behaviors, vaccination and post-COVID-19 perceptions, thus testing and extending their new insights. This session focused on skill development and, as such, to meet the learning objective – learning about analytical frameworks for systematically approaching contemporary public health issues in an international context – students shared their observations from interviews with family and friends and using the SDH framework, identified barriers and enablers of cross-cultural healthcare seeking behaviors at the individual, community, and system levels. In groups, students synthesized their findings as text into the SDH framework template using Google docs (see text footnote 2) as the collaborative platform, sharing collective reflections in the main room.

**Figure 2:**
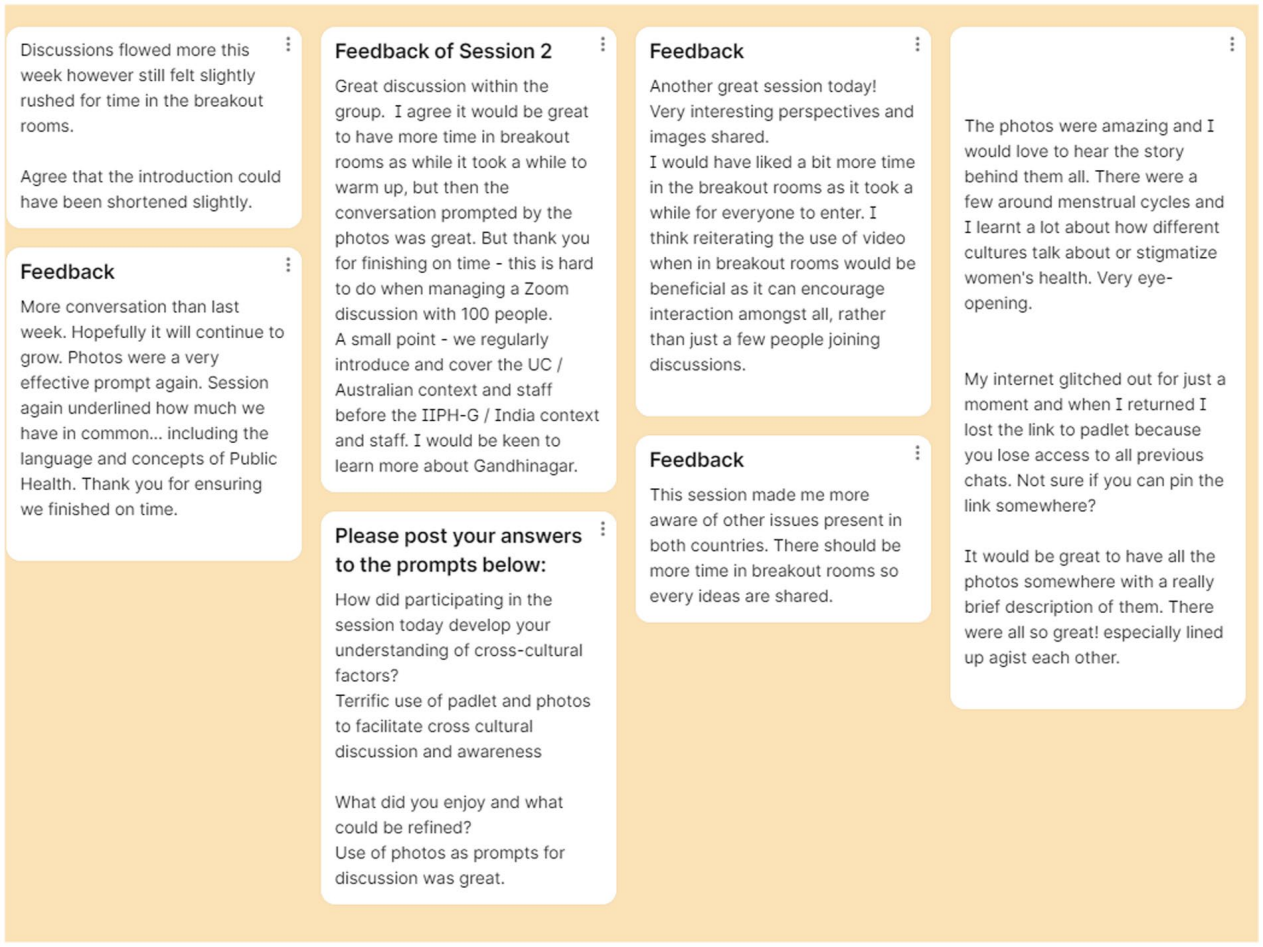
Example of post-session free text feedback provided by UC students using Padlet.

The third and final tutorial session was on applied focus. As such to meet the learning objective – apply theory and real-life experience to develop a health system intervention to address a contemporary global health challenge – students were required to prepare in advance of the session by reading two case studies that were identified and shared by study authors from the open access BMJ Global Health case study series (29, 30) which reflected global health-based cases suited to the session’s overall learning objectives. Students were asked to extract cultural and SDH insights from each case study and bring related photos they identified illustrated healthcare-seeking behaviors related to the case series in preparation for this session. In the session, mixed student groups were randomly allocated to one case study and were tasked with developing a (draft) E-poster on Padlet applying the SDH framework and exploring ways to improve health outcomes (see Figure 2). Each group produced and presented their collaborative work to all participants in the main room: demonstrating the use of online, collaborative tools, demonstrating the assimilation of new knowledge, skill development, and relationships, and the development of cultural insights over the 4 weeks. Groups presented their E-poster in the final Zoom main room to each other and to invited guests who represented senior UC and IIPH-G academics. This final session was a virtual simulation of the approach global health practitioners take in co-preparing and presenting healthcare interventions.

### 3.3. Learning outcomes and evaluation framework.

The learning outcomes were assessed in session by the completion of an E-poster forming visual evidence of the application of student learning, and formally using the Qualtrics survey at the end of the final session. The Qualtrics survey sought to capture the impact of participation in the program by adopting a validated International Education Survey (IES) questionnaire [31] that measured four key themes related to; “motivation,” “international perspective,” “personal development” and “intellectual development.” Evidence from the survey generated quantitative evidence to evaluate the impact of the program and complimented the free text qualitative information that was collected from students after each session *via* Padlet.

While these suggestions fed into iterative improvements for each session, the formal evaluation of the program comprised the application of learning evidenced by a co-produced E-poster which demonstrated facilitated reflection and meaning-making through the process of social interaction with others (32) (b) and the completion of Qualtrics Survey (c) in the final session. The three evaluations are detailed below.

a. In-session process evaluation: free text qualitative feedback (see Figure 2).

All students were provided dedicated feedback Padlet links at the end of each session with the option of providing feedback around session engagement and suggestions for improvement.
Both universities were separately provided with a Padlet link where they could provide optional feedback using open-text comments around prompt questions centered around engagement, learning outcomes and other suggestions. For each session, the feedback was reviewed by the study authors / facilitating academics and thus, subsequent sessions were refined to incorporate suggestions where feasible. This progressively enabled modifications of the sessions, including for and by each university student group.
b. In-session outcome evaluation: Creation of an E-poster.

**Figure 3:**
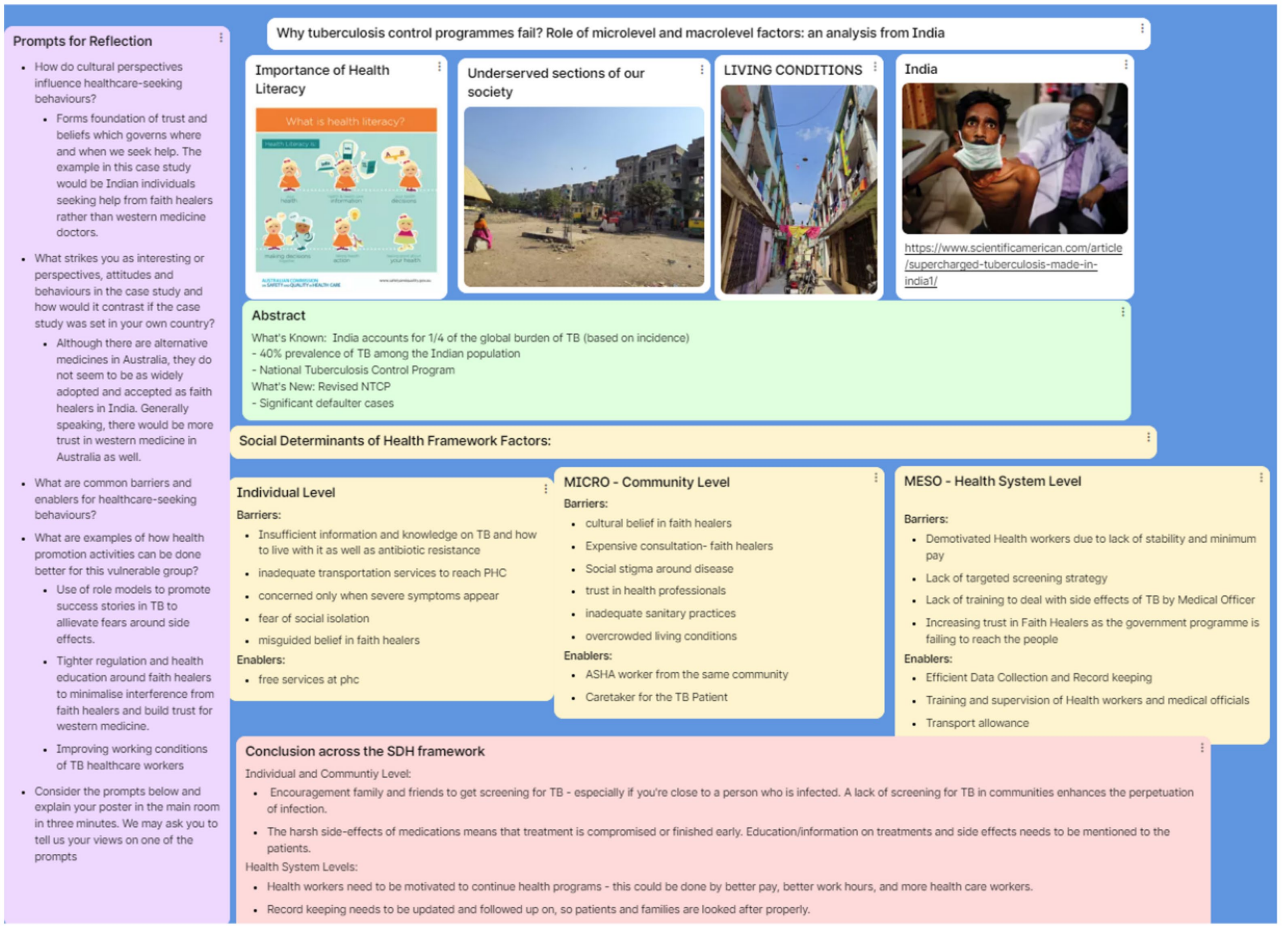
A completed E-poster on tuberculosis program management presented online by one of 10 student groups in the final I-PELICAN session.

The E-poster template was created by the study authors, using prompt questions linked to two contemporary global health case studies adapted from the BMJ’s case studies series (29, 30), which related to the Tuberculosis program management in India and Migrant health care in Australia. The E-poster template was novel in that it linked a global health case study to the social determinants of health framework but also allowed students to construct new meaning or knowledge virtually and collaboratively while determining a health promotion strategy. The E-poster tool was not only part of the assessment strategy but also provided visual evidence of students’ collaborative application of this new learning, which they used as a presentation aid in sharing and reflecting back to the whole cohort. The completion of the E-poster and the degree to which the students successfully completed the E-poster was an indicator of a successful curriculum and purposeful student engagement. As an illustration, Figure 3 in the result section 5.3 provides an example of a completed E-poster on Tuberculosis program management presented online by one of 10 student groups in the final I-PELICAN session.
c. In-session outcome evaluation: Modified International Education Service-Learning survey:
The main evaluation outcome was an online Qualtrics survey adapted from the IES questionnaire which was provided to all students and facilitators at the end of the final session in week four. This sought students’ self-assessment of their participation outcomes in the I-PELICAN series across four themes, namely “motivation,” “personal development,” “intellectual development,” and “international perspectives.” Previous studies have validated the scale reliability of the IES questionnaire and constituent themes (31, 33). The survey data were descriptively analyzed to identify means and standard deviation for the individual items and the four themes in Microsoft Excel version 2,301. Results from the Qualtrics survey across the themes are presented in this paper. The study authors also designed a special certificate of appreciation using graphic designing software CANVA (34) for students who completed three out of four sessions and completed the online Qualtrics survey to use as evidence of virtual work integrated learning participation.
Broadly the aim of the post-session evaluation was to determine:

- students’ motivations to participate in these sessions,
- to what extent their professional development and intellectual development was enriched by participating in the experience and the collaborative application of new learning evidenced by the completion of the E-poster as relevant to the case study they were assigned,
- if the experience enhanced/influenced their international perspectives,
- if students valued learning about cross-cultural influences in healthcare-seeking behaviors.

## 4. Results

For the purposes of this paper, the I-PELICAN session’s formal evaluation comprises the IES Qualtrics survey and the created E-posters. The results are provided across each of the domains of the Qualtrics survey, which are supplemented by free-flowing narrative feedback that students provided.

### 4.1. Modified International Education Survey findings

The demographic results of the students who participated in the end-of-session Qualtrics survey are provided in Table 1 below. In all, there were 102 students from both Australian and Indian universities combined, out of which the majority, 64 students (75%), were Indian students from IIPH-G. The sessions were attended by both undergraduate and postgraduate students from UC who were enrolled in the co-taught undergraduate /postgraduate Global Health unit, with more undergraduate students (17) participating compared to postgraduate students (8). Among these, most UC students were enrolled in the Bachelor of Physiotherapy course but taking the global health unit as an elective unit/subject, followed by MPH students. As IIPH-G specializes in postgraduate-only courses, overall, most participating students (68) came from the MPH course at IIPH-G. Additionally, the age and gender details showed that 68 students identified as female gender with a median age of 25 years across both universities. UC as a university focuses on enhancing equity and social inclusion in their student cohorts which was also seen in the wider age range of participating UC students (19–53 years), suggesting the presence of mature-age students in the UC participating cohort as well. The survey also showed that from a linguistic perspective, English was the most common language spoken at home for UC students, while in India, most students shared that they came from “Hindi” speaking households. The survey also enquired about international exposure through travel and living abroad from each cohort of Australian and Indian students separately. The results showed that more UC students (70%) had international exposure compared to Indian students (27%).

**Table 1:** Basic sociodemographic details of participating students overall and from each university.

			University	
	Variable	Total responses from both Universities	University of Canberra (UC)	Indian Institute of Public Health -Gandhinagar
			(*n* /%)	(*n* /%)
1	Number of Students Participating	102	21 (24%)	64 (75%)
2	**Level of students**
	Undergraduate	17	13 (76%)	–
	Postgraduate (MPH)	68	8 (38%)	68 (100%)
3	**Course enrolled in**
	Undergraduate		Bachelor of Physiotherapy	–
	Postgraduate		Master of Public Health	Master of Public Health
3	**Gender**
	Male	15	8 (38%)	7 (11%)
	Female	68	13 (62%)	55 (88%)
	Non-Binary/Third Gender	1	–	1
4	The median age in years	25.6 years	25 years	23 years
		(SD 4.6)	(SD 2.7)	(SD 8.3)
		Range (19–53)	Range (20–34)	Range (19–53)
5	Have Australian/Indian students lived or traveled outside of their respective countries?		Yes: 21 (70%) out of 30 student responses from UC	Yes, 17 (27%) out of 62 student responses from IIPH-G.
6	Preferred language to speak at home		English	Hindi (English was the second most common language)

Table 2 provides survey results from the adapted IES instrument across four key dimensions, namely “motivation,” “personal development,” “intellectual development” and “international perspectives.” While the final session reported 102 participants, the individual number of responses for each of the domains varied.

**Table 2:** Aggregated student results for the key domains of “Motivation,” “Personal Development,” “Intellectual Development,” and “International Perspectives” from modified international education survey.

Motivation: I decided to participate in this I-PELICAN session’	Responses (*n* = 73)	Average (out of 5)	SD	Score Range
1. To learn more about a different culture	73	4.60		2–5
2. To improve my employability	69	3.65	1	1–5
3. To experience learning in a different context	74	4.60	0.6	3–5
4. For personal growth	74	4.50	0.7	2–5
5. Because of a course completion requirement	59	3.20	1.4	1–5
6. Because it suits my learning style	54	3.80	1.2	1–5
7. Because a friend/lecturer/classmate mentioned it	66	3.80	1.3	1–5
8. Other	10	3.00	1.8	1–5
Grand Mean for Motivation		4.36	0.9	1–5
**Personal Development: To what extent has your I -PELICAN learning experience as a student**	**Responses (*n* = 85)**	**Average (out of 5)**	**SD**	**Score range**
1. To give me an idea of whether I am interested in studying overseas	68	3.88	1.1	1–5
2. Increased the likelihood that you would work in another country	68	3.97	1.0	2–5
3. Because experiential learning with peers is a more effective way to learn for me	57	4.19	1.0	2–5
4. I would be motivated to attend the next series of I-PELICAN sessions with another university	72	4.33	0.9	2–5
5. I would encourage other public health university students to participate in I-PELICAN tutorials in the future	70	4.34	0.8	1–5
6. I would prefer to attend more of I-PELICAN like sessions in place of regular global health classes	60	4.37	0.7	3–5
7. Facilitate an intercultural dimension in your volunteer activities	69	4.38	0.7	2–5
8. Contributed to your level of self-confidence to engage with cross-cultural peer learning	70	4.40	0.8	2–5
9. Enhanced your interaction with people from other cultures	69	4.40	0.7	2–5
*Grand Mean for Personal Development*		4.25	0.90	1–5
**Intellectual Development: To what extent has your I-PELICAN learning experience as a student**	**Responses (*n* = 85)**	**Average (out of 5)**	**SD**	**Score range**
1. Inspired you to read further on cross-cultural influences on global health topics	69	4.25	0.8	2–5
2. Inspired me to question assumptions when discussing cultural issues in global health	71	4.25	0.7	2–5
*Grand Mean for Intellectual Development*		4.25	0.80	2–5
**International Perspectives: To what extent has your I PELICAN learning experience as a student.**	**Responses (*n* = 85)**	**Average (out of 5)**	**SD**	**Score range**
1. Enhanced your understanding of Australian culture	72	3.96	1	1–5
2. Enhanced your understanding of Indian culture	70	4.50	0.8	2–5
3. Facilitated an international or intercultural dimension in your work or activities	70	4.27	0.8	2–5
4. Influenced your understanding of global health issues in other countries	73	4.29	0.8	1–5
5. Influenced your discussion with other people about international and transcultural issues	69	4.30	0.8	2–5
*Grand Mean for International Perspectives*		4.30	1.30	1–5

### 4.2. Modified International Education Survey results

Students reported an overall high mean impact of 4.29 (out of 5) across all four themes seen together. As shown in Table 2 below, the grand mean (*M*) was highest for the domain of “Motivation” (*M* = 4.36, SD = 0.9), followed by “International Perspectives” (*M* = 4.30, SD 1.30) and similar scores for Personal Development (*M* = 4.25, SD = 0.90), Intellectual Development (*M* = 4.25 SD = 1.30).

Individual items within domains showed means ranging from the lowest mean of 3.20 with regards to participating in the I-PELICAN sessions as a motivating factor to the highest mean of 4.60 for reasons related to learning more about a different culture and experiencing learning in a different context.

As shown in Table 2, the domain “Personal Development” reported the highest mean (*M* = 4.40) for the item responses “*Contributed to your level of self-confidence to engage with cross-cultural peer learning*” and “*Enhanced your interaction with people from other cultures*.” The lowest mean (*M* = 3.88) referred to “*give me an idea of whether I am interested in studying overseas*.” This is an encouraging result as it indicates that the planned cross-cultural learning environment worked as it was intended to provide students with a virtual experience of cross-cultural healthcare practices.

Regarding the domain of “Intellectual Development,” both items reported similar high mean scores of 4.25, reflecting the impact of participating in the I-PELICAN sessions on providing a greater awareness and interest in global health topics. In terms of the final domain of “International Perspectives,” the high overall mean was reported for an enhancement of Indian culture, this reflects both students from Australia and India jointly reporting that the sessions enhanced their worldview and understanding of how culture in India influenced health-seeking behaviors.

The above quantitative survey results represented the final outcome evaluation findings using the adapted IES instrument. Separately, insights from the final in-session E-poster activity reflecting the applied focus or skill development aim of the I-PELICAN session are presented.

### 4.3. Insights from the in-session E-poster activity

The final session represented the “applied focus” of the I-PELICAN sessions, where cross-cultural groups of students were provided with the opportunity to apply the SDH framework to identify and develop a new health promotion strategy using the E-poster template. Successful poster creation and in-session presentation were reflective of successful engagement and cross-cultural knowledge development across the I-PELICAN sessions. In the final session, all (ten) groups completed the E-poster which also validated its usability in a cross-cultural and mixed student setting. As outlined, the IPELICAN sessions foci were to encourage new knowledge and ways of thinking about health promotion activities, and this aspect was observed *via* the quality of the E-posters produced and student engagement in the sessions that were jointly observed by the study authors. As an illustration, a co-produced E-poster from one group is provided in Figure 3. Student engagement showed that structured prompts were meaningful to drive the discussion while providing flexibility in addressing the questions. Separately student’s Padlet feedback as seen below showed an appreciation for a supportive online environment that fostered engagement by linking cultural immersion through sharing photographs and cultural stories as evidenced *via* the completed E-posters.

I really liked this way of learning through pictures, we got to learn a lot about each other and our ways of thinking. Not only do we just share our photos, the discussion after is interactive. Each photo shows the students’ effort to bring different aspects of life to the class. It keeps us motivated to do better by next class. (IIPH-G, India student respondent).

While the E-poster activity was mainly reviewed in-session, students’ experience of participation also came through a separate post-session narrative written reflection using Borton’s framework (34), which Australian students completed as part of an in-unit assessment. For non-participating UC students, an alternative reflection was created and compared by study authors, but for the scope of this paper, only key reflections from participating students around themes of peer-to-peer engagement, cultural appreciation, and learning are presented. Students indicated that they appreciated the virtual cross-cultural learning experience.

the experience of presenting to a global cohort cannot be understated and was a significant learning point for myself.

The deliberate use of cooperative learning groups served to foster social interactions (9) for cross-cultural sharing and learning. Although it is recognized that there are some benefits in mixing up student groups, keeping the groups the same over such a short timeframe and with the intention of building social trust to enhance sharing and contributions proved to be essential.

…the biggest strength noticed by myself and others in my group was the fact that we remained with the same students throughout our entire global experience. Because of this, we were able to build trust and friendships in order to speak about things that may have been harder to talk about with new students every week.

The student reflections also provided insights into the constructivist learning paradigm that informed much of the I-PELICAN session design. The sessions embedded constructs fostering peer-to-peer learning as a precursor for working in multicultural teams for future health care workers as well as recognizing the importance of understanding culture from a local lens or construct for designing health intervention. Student reflections indicated an appreciation of peer-to-peer engagement in an authentic manner, as shown by comments such as:

What stood out was the effect of cultural beliefs on healthcare. I was aware that culture has a great influence on health, but just how much, I never realized until these sessions. The interaction with the facilitators and other participants was brilliant. Everyone was rich with experience and happily and respectfully contributed to the discussions. About myself, I realized that I had been until now looking at health clinically, henceforth I need to look at it from a different perspective.

Overall, students indicated that participation in the I-PELICAN was a rewarding experience;

I was surprised to learn how different we were due mostly to the culture of India having a basis more in spirituality then our Australian culture. I was told that it’s not uncommon for families to use faith healers or similar figures to resolve common ailments – this can become problematic as with the case of TB.

## 5. Discussion on the practical implications, objectives, and lessons learned

### 5.1. Practical implications

The four I-PELICAN sessions were the first virtual global health series conducted between an Australian and Indian Public Health university, to our knowledge, where constructive learning pedagogy was embedded, utilizing a contemporary global health framework (SDH) to cross-culturally analyze real-world health case studies. The sessions were designed and delivered as a proof of concept, which demonstrated implementation feasibility and a high degree of student satisfaction, as evidenced by the IES survey and student results. The implementation feasibility is important to highlight here as the learning experience and methodology allow potential replication across other public health subjects among partnering universities but also serve as a template for other universities elsewhere. While online learning has transformed tertiary education and enhanced student equity, classical learning theory informs much of current, contemporary online learning approaches (4, 5, 9, 10). Our study adds to existing research and presents a novel method to embed constructivist learning theory and the application of a global health skills-based framework, the SDH, to support culturally and contextually relevant health promotion strategy development among future health workers (8, 11, 17, 18, 23). While the I-PELICAN sessions were first conceptualized as part of a teaching and learning grant awarded by UC to the study authors, it showed that cross-institutional support to explore teaching research is necessary for academics, especially at the early career stage as the first author to develop competencies in this area. Overall, the online module enhanced “global mindedness” for UC and IIPH-G students and strengthens UC’s position as a recognized international knowledge hub in Public Health. The subsequent sections provide an overview of salient themes.

### 5.2. Implementation insights

While over 100 students jointly participated in the four sessions over 4 weeks during August –September 2022, the sessions took approximately a month of lead-up time for planning, coordination and template design, which included pedagogy-focused workshop sessions jointly attended by UC and IIPH-G academics, where a teaching and learning pedagogy expert provided strategic guidance. Thus, the sessions included an element of capacity building for participating staff who developed virtual cooperative learning group facilitation skills and virtual pedagogy thinking as evidenced by staff feedback in post-session debriefing sessions and in the IES survey findings.

From an implementation perspective, the four sessions were conducted over ten hours in total, with each session scheduled for two hours, having predesigned templates for each session with embedded links for individual Zoom break-out rooms and Padlets, which was important for time management and collaborative access. However, student feedback indicated that more group time would be advantageous for each of the sessions, and therefore, the session plans were adjusted accordingly. The templates were developed by the study authors after the pedagogy workshop and using a facilitative process. UC has a focus on enhancing student equity in university participation, and the I-PELICAN series intrinsically wove student equity in the design to allow greater student participation. In addition to these drivers, one of the underlying principles was being able to demonstrate the proof of concept with minimal financial investment to the participating students and universities involved. As an example, the use of Zoom and Padlet for which institutional licenses were used, allowed technology to be leveraged that was already being used by both institutions and therefore did not require added training time for students. Moreover, by embedding the sessions in the semester as part of an existing global health unit/subject at UC and IIPH-G, attending the sessions was facilitative for students and did not create additional time commitments.

### 5.3. Foster cross-cultural appreciation through peer-to-peer engagement

An important component of the I-PELICAN session was facilitating intercultural interactions among students early on, using in-session activities such as photograph sharing and observation sharing of health care seeking interviews of family members and others. These activities helped to develop an applied understanding of how cultural perceptions are shaped in respective countries beyond theoretical concepts and actively demonstrated how they influence healthcare-seeking behaviors. By starting the series with a cultural immersion activity where students were encouraged to bring photographs depicting local culture across themes of place, person, and healthcare settings, students showcased to each other the cultural constructs between the two countries and also supported learning in an open and respectful manner.

### 5.4. Enhancing online social connections

While online sessions have enhanced the delivery of educational content, Greenan et al. (35) noted challenges in online teaching compared to traditional classroom teaching in fostering socialization and student communication (35). Yet studies also note that social connections can still be fostered in online sessions through educators being available, positive, and sympathetic in creating online environments where students have increased opportunities to exhibit social presence or a sense of being together (1, 14–17, 25, 26, 36). The I-PELCIAN sessions sought to encourage social connections among students through individual activities in each session but also structuring safe, student-friendly online spaces in the break-out rooms where students could engage with each other and have greater opportunities for authentic self-disclosure and engagement. A sense of togetherness or social presence was also conveyed through processes of collective reflections and insight generation in separate groups over individual students’ ideas in the sessions. Students, for example, used photographs in session one and individual interviews collected from family and friends on COVID-19 experiences in the second session to share insights and co-create new knowledge and understanding of cultural barriers and enablers to health care-seeking behaviors. Photographs that students displayed in individual break-out rooms using the group’s Padlet were then voted upon collectively to identify two–three representative photos to bring back to the main room to share with all participants. By structuring each activity through a sequence of transitions between the main room and break-out rooms (for individual group discussions) and back to the main room for presentations and reflections to the whole cohort, students have presented an opportunity to construct their own understanding in smaller groups and engage with peers in an open, supportive non-judgmental virtual space with academic facilitators in the background as support if needed. This created a “safe space” for students to respectfully work together – simulating real-world, virtual, cross-cultural, collaborative PH environments. While also accommodating flexibility in attendance, maintaining the same mixed-group allocation of IIPGH and UC students over the sessions allowed for better cohesion, engagement, and consistency across sessions as validated by student feedback.

In conclusion, the I-PELICAN sessions that were developed as a proof of concept showcased implementation feasibility and provided learnings to allow flexible integration in other core Public Health units and expansion to other universities in the future. The online sessions provide a novel means to enhance the global health unit offerings with a greater focus on cultural awareness and skill development at universities and provide greater equity for students in accessing global mobility-aligned opportunities at no extra cost to a regular unit enrolment fee. It also provides an opportunity to enhance student equity by being available to students regardless of the opportunity to do in-country placements and providing flexibility for students who have competing work, and family caregiving commitments.

## Data availability statement

The raw data supporting the conclusions of this article will be made available by the authors, without undue reservation.

## Ethics statement

The studies involving human participants were reviewed and approved by Human Research Ethics Committee, University of Canberra. The patients/participants provided their written informed consent to participate in this study.

## Author contributions

DA, RM, and JS conceptualized, implemented and evaluated the I-PELICAN sessions with support from SS, DS, and DM. DA and RM conceptualized the evaluation framework and analyzed the data for the process and final outcomes. JS provided pedagogy expertise for session design and delivery and contributed to manuscript drafting and editing. DA, RM, JS, DS, SS, and DM engaged in editing the manuscript. All authors contributed to the article and approved the submitted version.

## Funding

The I-PELICAN sessions were conducted under the University of Canberra’s Faculty of Health Teaching and Education Research Grant (TIGER)2022. No expenses were, however, committed during any stage of the I-PELICAN sessions.

## Conflict of interest

The authors declare that the research was conducted in the absence of any commercial or financial relationships that could be construed as a potential conflict of interest.

## Publisher’s note

All claims expressed in this article are solely those of the authors and do not necessarily represent those of their affiliated organizations, or those of the publisher, the editors and the reviewers. Any product that may be evaluated in this article, or claim that may be made by its manufacturer, is not guaranteed or endorsed by the publisher.

## Footnotes

^1^ https://www.padlet.com

^2^ https://www.google.com.au/docs
